# Synthesis of Silver Nanoparticles Using Hydroxyl Functionalized Ionic Liquids and Their Antimicrobial Activity

**DOI:** 10.3390/ijms9050807

**Published:** 2008-05-20

**Authors:** Demberelnyamba Dorjnamjin, Maamaa Ariunaa, Young Key Shim

**Affiliations:** School of Nano System Engineering, Inje University, Kimhae, 621-749, Korea 302-375. Website: http://homepage.inje.ac.kr/~nano/

**Keywords:** Silver nanoparticles, hydroxyl functionalized ionic liquids, hydroxyl functionalized cationic surfactants, antimicrobial activity

## Abstract

We report a new one phase method for the synthesis of uniform monodisperse crystalline Ag nanoparticles in aqueous systems that has been developed by using newly synthesized mono and dihydroxylated ionic liquids and cationic surfactants based on 1,3-disubstituted imidazolium cations and halogens anions. The hydroxyl functionalized ionic liquids (HFILs) and hydroxyl functionalized cationic surfactants (HFCSs) also simultaneously acts both as the reductant and protective agent. By changing the carbon chain length, alcohol structure and anion of the 1,3-imidazolium based HFILs and HFCSs the particle size, uniform and dispersibility of nanoparticles in aqueous solvents could be controlled. Transmission electron microscopy (TEM), electron diffraction, UV-Vis and NMR, were used for characterization of HFILs, HFCSs and silver nanoparticles. TEM studies on the solution showed representative spherical silver nanoparticles with average sizes 2–8 nm, particularly 2.2 nm and 4.5 nm in size range and reasonable narrow particle size distributions (SD-standard distribution) 0.2 nm and 0.5 nm respectively. The all metal nanoparticles are single crystals with face centered cubic (fcc) structure. The silver nanoparticles surface of plasmon resonance band (λ_max_) around 420 nm broadened and little moved to the long wavelength region that indicating the formation of silver nanoparticles dispersion with broad absorption around infrared (IR) region. Silver complexes of these HFILs as well as different silver nanoparticles dispersions have been tested *in vitro* against several gram positive and gram negative bacteria and fungus. The silver nanoparticles providing environmentally friendly and high antimicrobial activity agents.

## 1. Introduction

There has been an increased awareness about the role of contaminated environmental surfaces within the home and health care settings as a potential vector of disease transmission. Many disinfectants contain nonvolatile antimicrobial agents such as quaternary ammonium compounds (QACs) that can leave an antimicrobial residue on treated surfaces. The potential of these agents to prevent bacterial colonization is limited because of their lack of persistence on surfaces after some environmental insult, such as water contact or rubbing. These moist environments and physically contacted surfaces are the most likely to be contaminated, to allow bacterial proliferation, and to act as a pathogenic reservoir. For a given disinfectant technology to realize a significant residual antimicrobial benefit, it must persist under such conditions.

A unique new silver based material has been shown to provide a persistent antimicrobial benefit that can provide environmental control of pathogenic bacteria on treated surfaces. Research showing colloidal silver's superior performance in fighting multiple disease causing pathogenic microorganisms including anthrax has attracted the attention of leading scientists, given the current concerns over terrorism. Colloidal silver have been known for a long time to possess antimicrobial properties and also to be non-toxic and environmentally friendly. Silver is used as a biocide to sterilize recycled drinking water aboard of the NASA space shuttle and MIR space station. In fact, the real gold standard among antimicrobial agents turns out to be not any type of silver, but a new, patent pending form of stabilized colloidal silver particles that kills virtually any germ [1–2].

Silver may have an important advantage over conventional antibiotics in that it kills all pathogenic microorganisms, and no organism has ever been reported to readily develop resistance to it. Researchers believe that the potential of colloid silver is just beginning to be discovered.

Nanoparticles have been extensively investigated due to the attraction of their unique physical properties, chemical reactivity, and potential applications with high academic and industrial impacts [3–5]. Usually when metal nanoparticles are prepared by chemical methods, the metal ions reduced by the reducing agents and a protective agents or phase transfer agents are also added to stabilize the nanoparticles [6–8]. Several types of toxic reducing agents containing boron commonly have been employed to produce metal nanoparticles from inorganic salts, the resulting metal nanoparticles are contaminated with borides. This allows for the preparation of boride free metal nanopowders specially for use in biological and medical purposes.

The use of quaternary ammonium for stabilizing metal nanoparticles was proposed by Bonnemann [6] and the particles are not dispersible in water because their anionic particle's surface is surrounded by the hydrophobic tetraoctyl quaternary ammonium ions. Particles synthesized in organic solvents are water-immiscible, which limits their applicability. Many applications require for nanoparticles to be dispersible and stable in water [9]. However, water-based synthesis of nanoparticles are fraught with many problems such as ionic interactions, low reactant concentration and stabilizers are difficult to remove [10]. The particles synthesized in organic solvents can be made at relatively high concentrations [11], with predefined size and shape and with improved monodispersity [12] when compared with those prepared in aqueous media.

Here we first used mono and dihydroxyl functionalized ionic liquids (ILs) and cationic surfactants for preparation silver nanoparticles can be readily dispersed into water. The formation and stabilization efficiency values of HFILs and HFCSs can be tailored by varying the ring structure, the substituting groups in the cations and the counter anions. The anions also plays a key role in formation and stabilization of nanoparticles and can be rationalized by considering the difference in the anion size of surfactants. Based on the same principle, we also envision the potential uses of HFILs and HFCSs containing different functional groups as selective reagents used for the formation and stabilization of nanoparticles.

In this research, we synthesized Ag nanoparticles by using newly synthesized mono and dihydroxyl functionalized ILs and cationic surfactants. These ILs and surfactants were designed to have one, and two alcohol groups, different long chain structure, the former serving to produce small nanoparticles with different sizes dependence of number of alcohol groups, chain length and their position in molecules. Herein, we report our preliminary results on the preparation of novel ionic liquids and cationic surfactants terminated alcohol ligands in cation and one pot synthesis of Ag nanoparticles stabilized by these ligands, their antimicrobial properties.

## 2. Results and Discussion

**Figure 1.**
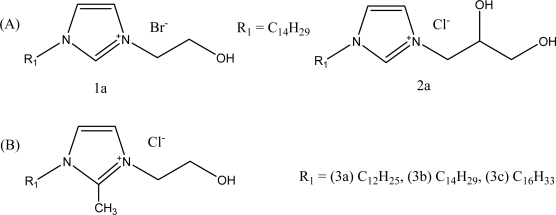
Molecular structures of hydroxyl functionalized ILs and surfactants.

First of all, as initial attempts, two different series of quaternary imidazolium salts (alcohol ionic liquids and surfactants) were synthesized: series A: 1-alkyl-3-hydroxyethylimidazolium bromide, 1-alkyl-3-(2’,3’-dihydroxypropyl)imidazolium chloride; series B:1-alkyl-2-methyl-3-hydroxyethyl-imidazolium chlorides. Their structures are shown in Figure 1.

**Scheme 1.**
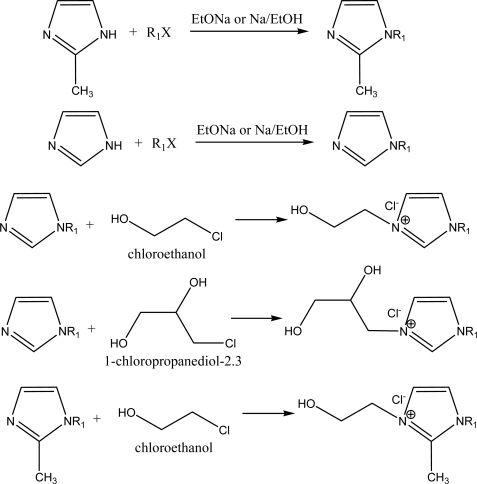
Synthesis of hydroxylated ionic liquids.

For the synthesis of these alcohol ionic surfactants, we used 1-alkylimidazole, 2-methylimidazole, 2-chloroethanol and 1-chloro-2,3-propandiol and they were easily prepared in two steps using standard procedures [13–14], as shown in Scheme 1. The novel dihydroxyl functionalized cationic surfactant 1-tetradecyl-3-(2’,3’-dihydroxypropyl)imidazolium chloride was easily prepared from 1-tetradecylimidazole and 1-chloro-2,3-propanediol. We synthesized five salts based on various types of cations made by changing the carbon number of alkyl substituents in order to establish the influence of chemical structure on surface activity.

The salts were obtained by deprotonation of imidazole by sodium or sodium ethanoate followed by alkylation in ethanol or acetonitrile. Alkylation of the 1-alkylimidazole derivatives were conducted by refluxing in methanol or acetonitrile a solvent chosen for its stability toward strongly alkylating agents, its moderately high boiling point, and the insolubility of the imidazolium salts in this medium. The longer chain salts are low melting crystalline solids the shorter chain salts are liquids in ambient temperature.

We tested silver nanoparticles prepared by the following quaternary 1-alkyl-3-hydroxyethylimidazolium compounds: (A), 1-hydroxyethyl-3-tetradecylimidazolium bromide (C_14_HEtImBr, **1a**); 1-(2’,3’-dihydroxy)propyl-3-tetradecylimidazolium chloride (C_14_diHPrImCl, **2a**); (B),1-hydroxyethyl-2-methyl-3-dodecylimidazolium chloride (C_12_HEtMeIm, **3a**); 1-hydroxyethyl-2- methyl-3-tetradecylimidazolium chloride (C_14_HEtMeImCl, **3b**), and 1-hexadecyl-3-hydroxyethyl-2-methylimidazolium chloride (C_16_HEtMeImCl, **3c**). All these imidazolium chlorides and bromides are highly surface active agents that have two hydrophobic alkyl substituents in the 1 and 3 positions or 1 and 2 positions in the imidazolium ring.

A preliminary investigation of the antibacterial and antifungal activities of HFILs and surfactants was performed through measurements of minimal inhibitory concentrations (MIC) expressed in μg/mL based on our previous study [15]. The efficiency of Ag nanoparticles stabilized by these salts were evaluated against bacterial and fungal strains through measurements of minimal inhibitory concentrations (MIC) expressed in μg /ml, values after one day of exposure are shown in Table 1. The 2 series of A:1-hydroxyethyl-3-tetradecylimidazolium bromide (C_14_HEtImBr, 1a),1-(2’,3’- dihydroxy)propyl-3-tetradecylimidazolium chloride (C_14_diHPrImCl, **2a**); and series B: 1-hexadecyl-3-hydroxyethyl-2-methylimidazolium chloride (C_16_HEtMeImCl, **3c**) quaternary salts stabilized Ag nanoparticles were the most efficient salts among these tested. The two series of quaternary salts stabilized Ag nanoparticles showed the broadest bactericidial and fungicidal activity.

The more important of the two series was the 1-hydroxyethyl-3-tetradecylimidazolium bromide (C_14_HEtImBr, **1a**)(A) salts. Their antibacterial and antifungal activity of Ag nanoparticles was greatly affected by surfactant stabilizers chain length, the type of substituted functional groups, and their position in the imidazolium ring.

The variation of these factors can lead to high activity compounds (Series A and B). For the tested series B quaternary imidazolium compounds, we found the preferred 1-alkyl group's chain length for the controlling test organisms to be in the C_12_ to C_16_ range. The results show all of the tested imidazolium halides stabilized Ag nanoparticles that have one hydrophobic and one hydrophilic group in the 1 and 3 positions in the imidazolium ring were the most suitable for antimicrobial activity. Also, the long alkyl chain imidazolium salts with methyl and hydroxyethyl substitution in the 2 and 3 positions of the imidazolium ring have an efficient antimicrobial activity.

Seven microorganisms were chosen as test strains: *Bacillus subtilis* KCTC1914, *Staphylococcus aureus* 209 KCTC1916, and antibiotic resistant S*taphylococcus aureus* R209 KCTC1928 among gram positive bacteria, *Escherichia coli* KCTC1924, *Salmonella thypimurium* KCTC1940, among gram negative bacteria, and *Candida albicans* KCTC1940 as a representative fungus.

In view of the results, it appeared that the all tested quaternary imidazolium salts stabilized Ag nanoparticles are the most effective products against the tested bacterial and fungal strains. We evaluated the antimicrobial activities of these series compared them with commercially produced and widely used quaternary ammonium substances (benzalkonium chloride and cetylpyridinium chloride) and antibiotics (Gentamycin and Kanamycin).

**Figure 2.**
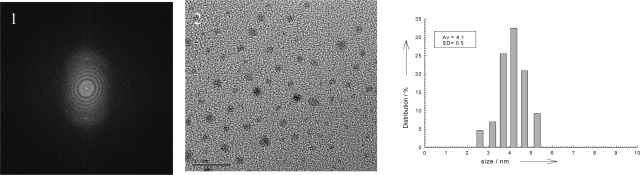
Electron diffraction (1) and TEM (2) images and histogram of silver nanoparticles stabilized by (C_12_HEtMeIm, **3a**) bar length 20 nm.

**Figure 3.**
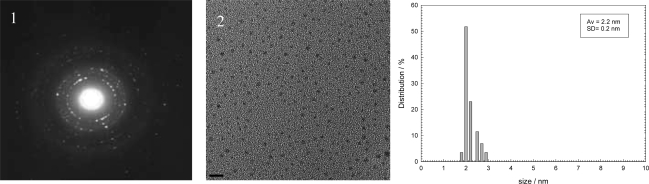
Electron diffraction (1) and TEM (2) images and histogram of silver nanoparticles stabilized by (C_16_HEtMeImCl, **3c**), bar length 10 nm.

**Figure 4.**
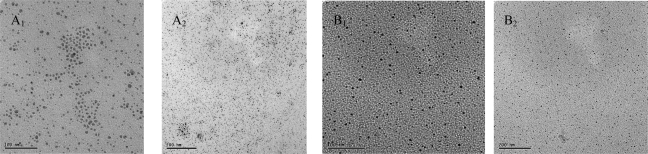
TEM high and low magnification images of silver nanoparticles (A_1_, A_2_ and B_1_, B_2_) stabilized by (C_14_diHPrImC_l_, **2a**) and (C_14_HEtImBr, **1a**) bar length 200 and 100 nm.

The TEM images for the HFILs coated Ag nanoparticles are shown in Figures 2–4 which display a representative selection of the crude nearly spherical nanoparticles. The silver nanoparticles as shown in histogram are small, average size of and 4.5 nm (C_12_HEtMeIm, **3a**), 2.2 nm (C_16_HEtMeImCl, **3c**), with reasonable uniform narrow size distributions 0.5 nm, 0.2 nm respectively.

The silver nanoparticles stabilized by (C_14_HEtImBr, **1a**) and (C_14_diHPrImCl, **2a**) as shown in Figure 4 are also small, uniform sizes of 4–8 nm and 2–4 nm respectively. The images can be seen that the silver nanoparticles are well separated, with no apparent sign of aggregation.

A representative detailed section of the electron diffraction images in Figure 2 and 3 further corroborate the crystalline nature of these metals nanoparticles. At this higher magnifications the crystalline nature of the metal cores are visible by the appearance of lattice fringes. The electron diffraction images in Figure 2 and 3 consist of discrete layers which identical to a lattice spacing {1.1.1} of bulk metal.

Electron diffraction reveals that all silver nanoparticles are crystalline, with face centered cubic (fcc) packing arrangements of bulk metals. The standard deviation are rather small, indicating that very efficient coordination of stabilizer- alcohol-ionic liquids to Ag (0) atoms or its prevents formation of larger Ag (0) aggregates.

The metal nanoparticles obtained here appear too small to show possible that the strong coordination of alcohol and diol's oxygen “O” and imidazolium carbon “C” in position 2 to the metals. HFILs as an ion compound can lead to easy solvation and stabilization of a metal salt in the water phase in that the interaction between IL and metal salt overcome the interaction between the water and metal salt [16]. [RImCH_2_CH_2_OH][X] functions as a reducing agent which reduces the Ag ion to Ag^0^ as well as a stabilizer in forming Ag nanoparticles.

(1) 2Ag+  +  [R−Im−CH2CH2OH]X  →  2Ag0  +  [R−Im−CH2CHO]X  +  2H+

It is thought that the –CH_2_OH- group in [RImCH_2_CH_2_OH][X] is converted to the –CHO- group by reducing Ag ion and protects the Ag nanoparticles at the same time according to the reaction equation 1 [17–18]. In FT-IR spectra, a new peak assigned to a frequency of a carbonyl stretching band appeared at a lower frequency, 1637 cm^−1^, for the reaction solution of IL-Ag suggesting some interaction between the carbonyl groups and ion Ag^+^ [18].

**Figure 5.**
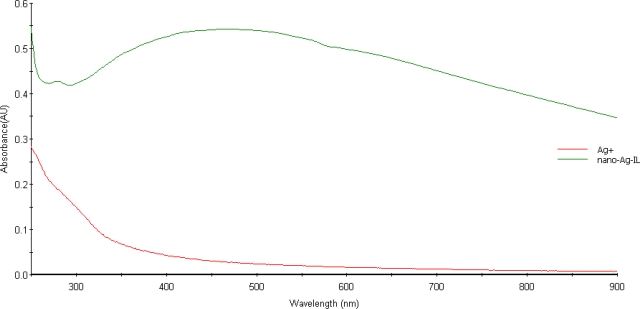
UV-Vis absorption spectrum of ionic silver (red line) and typical surface plasmon absorption spectrum of silver nanoparticles stabilized by HFIL: [C_12_H_25_HEMIm][Cl] (green line).

One finding is that the size and distribution of nanoparticles depends strongly on the length of side chains and structure of alcohol hydroxyl groups of the cations. From the viewpoint of the alky chain length, Figure 2 and 3 shows both the average size and standard deviation become smaller in the order of [C_16_HEMIm][Cl] > [C_12_HEMIm][Cl], which suggest that the slow growth rate of Ag nanoparticles by [C_16_HEMIm][Cl] results in small and mono-dispersed Ag nanoparticles [17]. From the viewpoint of the alcohol hydroxyl functional groups, as showed TEM images of silver nanoparticles in Figure 5 the size of nanoparticles become smaller in the order of (C_14_HEtImBr, **1a**) > (C_14_diHPrImCl, **2a**). Compare with silver nanoparticles stabiled by the di- hydroxyl alcohol group containing (C_14_diHPrImCl, **2a**), the silver nanoparticles stabilized by mono hydroxyl alcohol group containing IL: (C_14_HEtImBr, **1a**) were most a high antibacterial activity among tested ILs.

Comparison of the results of ^1^H-NMR measurement of pure ionic liquids and Ag nanopartilcles reveals substantial broadening of all the alcohol group related signals from ionic surfactants, when passivating the Ag nanoparticles. In the NMR traces of the reduced Ag nanoparticles the complete disappearance of the numerous peaks in the aromatic and aliphatic regions could be observed. In particular, the resonances of the alcohol protons and protons on the carbon α to the alcohol groups in cation are broadened to a such at extent to be no longer visible. This indicates that alcohol groups in ionic surfactants are in close contact in with the metal's surface with the hydrocarbon chains pointing outwards. Also, treatment of the Ag salt with hydroxyl functionalized imidazolium ionic liquids at room temperature afforded the corresponding diaminocarbene. This reaction was expected to produce Ag-carbene adducts at the surface of the nanoparticles. Free diaminocarbenes are described as better donors than the best phosphine donors [19–20] and strongly coordinated with these metals. The ^1^H- NMR spectrum of silver nanoparticles stabilized by (C_14_HEtImBr, **1a**) displays no H^2^ signal in the position 2 in imidazolium ring indicating deprotonation by metals in this position. The long range coupling between the H^4^ and H^5^ ring protons in the nanoparticles are also no longer visible. Results were showed a negligible amount of ILs at the surface of the silver nanoparticle. Therefore, only the silver nanoparticles itself being able to comes into direct contact with the bacteria.

UV-visible spectroscopy is one of the most widely used techniques for structural characterization of silver nanoparticles. The UV-Vis absorption spectrum of [C_12_H_25_HEMIm][Cl] stabilized pink colored silver nanoparticles showed that after the self reduction of the silver ions by hydroxyl group of HFIL stabilizes the surface of the plasmon resonance (SPR) band (λmax) around 420 nm broadened and slightly moved to the long wavelength region, indicating the presence and formation of spherical silver nanoparticles. The optical absorption spectra of metal nanoparticles are dominated by surface plasmon resonances (SPR), which shift to longer wavelengths with increasing particle size. The position and shape of plasmon absorption of silver nanoclusters are strongly dependent on the particle size, dielectric medium, and surface-adsorbed species. The surface plasmon absorption of silver nanoparticles have the short wavelength band in the visible region around 420 nm is due to the transverse electronic oscillation.

Many ionic liquid and ionic surfactant type of structure are also present in natural and synthetic compound molecules. Only problem has been asked of the possible toxicology of ionic liquid and ionic surfactant (IS). According to our IL and IS research the ability to finely adjust the biological properties of ionic liquids and surfactants by changing their anion/cation combination is a real benefit. ILs and Ionic surfactants are entirely appropriate and applicable to the field of preparation of silver nanoparticles for screening of cancer cell [21].

Recent literature data reports encouraging results about the bactericidal activity of silver nanoparticles of either a simple or composite nature [22–23]. The mechanism of attack of the silver nanoparticles against bacteria do not known well. Energy-filtering transmission electron microscopy (EF-TEM) images [24] revealed considerable changes in the cell membranes upon treatment, resulting in cell death. Elechiguerra and coworkers [25] found that silver nanoparticles undergo a size-dependent interaction with human immunodeficiency virus type 1, preferably via binding to gp120 glycoprotein knobs. The silver nanoparticles undergo a size-dependent interaction with HIV-1, Hepatite C with nanoparticles exclusively in the range of 1–10 nm attached to the virus.

The alcohol-ionic liquid surrounding the nanoparticle surface and act as effective stabilizers of Ag nanoparticles which are became highly dispersible in aqueous media. The nanoparticle size and stability were effected by the structure, position and quantity of alchol groups of the stabilizer-ionic liquids and therefore chemical and physical interaction between the ionic liquids and metals plays a decisive role in determining the nanoparticles structure. The evaluation of antibacterial and antifungal surfactant structures leads us to consider several molecular parameters. The most important parameters were the introduction of a long alkyl chain, and the hydroxyethyl and methyl groups in different positions within the imidazolium. The most efficient structures were 1-alkyl-3-hydroxyethyl-imidazolium chlorides, bromides,1-alkyl-3-dihydroxypropyl chlorides, and 1-alkyl-3-hydroxyethyl-2-methylimidazolium chlorides with a long alkyl chain. Some of these Ag nanoparticles gave results globally superior to the commercially available products benzalkonium chloride (BAC) and cetylpyridinium chloride (CPC), used as references. Reduction of silver nitrate in mono and di hydroxylated cationic imidazolium surfactants proven to be a suitable method for the syntsesis of silver monodisperse particles in the nanometer size range. In this method the new surfactants acts both as reducing agent and medium growth of the metal nanoparticles. In order to control the growth step and then particle size, morphology was used chain size, number and different position of hydroxyl groups in cation of surfactants. Using the structure and activity correlation that we have established as a starting point, we are now able to synthesize variations of well defined structure parameters in order to optimize quaternary onium compounds with different heterocyclic ring structures for future development of new silver antiseptics and disinfectants.

In summary we have demonstrated for the first time that the alcohol-functionalized cationic surfactants and ILs are a suitable medium for the preparation and stabilization of silver metal nanoparticles, which sizes depending from the number of alcohol group and other groups their position in cation of ionic liquids.

The structural control of nanoparticles, and structural tuning of alcohol ionic liquids and cationic surfactant stabilizers provides a powerful strategy for the preparation of metal nanomaterials.

## 3. Experimental Section

### 3.1 Synthesis and Structure of Quaternary Imidazolium Compounds (QICs)

#### 3.1.1. Preparation of 1-alkyl-2-methylimidazoles

Sodium (2.3 g/0.1 mol) was dissolved in anhydrous ethanol (200 mL) and mixed with imidazole or 2-methylimidazole (0.1 mol). Then, the appropriate amount of alkyl chloride or alkyl bromide (0.12 mol) was added and the mixture was refluxed for 6 h. The precipitated sodium chlorides or bromides were filtered and further ethanol was removed by rotary distillation. The resulting yields were in the range of 70–80%.

#### 3.1.2. Preparation of quaternary imidazolium salts

Methylimidazole (0.1mol) or 1-alkyl-2-methylimidazole (0.1 mol) were dissolved in anhydrous acetonitrile and then alkyl halide (0.12 mol) was added. The reaction was carried out for 6 h under refluxing and the product purified by extraction with hexane and recrystallized from acetonitrile. The yields were 83–88%. The purities of all synthesized imidazolium salts were checked by ^1^H-NMR (Bruker AMX FT 500 MHz NMR spectrometer) and Mass Spectrometry (FAB Mass JMS-HX110A) after drying under high vacuum (10^−2^ Torr) at 70 °C for 12 h. The resulting chemical shifts and ion mass (*m/z*) are summarized in Tables 2–5.

#### 3.1.3. Preparation of silver nanoparticles

The one phase preparation of alcohol-functionalized Ag nanoparticles in water using hydroxylated cationic surfactants was straightforward and purification of nanoparticles was carried out under ambient conditions. A 0.01 mmol sample of alcohol-ionic surfactant (**1**) in water (10 mL) was added dropwise under vigorous stirring to an aqueous solution of AgNO_3_ (0.01 mmol). The reaction mixture was stirred for approximately 2 h at 60°C temperature. The mixture of Ag salt and ionic surfactants turned a different color, depending on the size of the silver nanoparticles. Part of the water was removed from the solution mixture and dry ethanol was added. The nanoparticles were precipitated, and the supernatant liquid was removed. The precipitate was washed in dry ethanol, and then centrifuged. The process was repeated several times until all starting reagents removed. The nanoparticles were dried in vacuum oven (0.04 Torr) at room temperature. The purified powder of Ag nanoparticles could be kept in powder form or redispersed into water quite readily and stable and no flocculates and precipitates were found. This indicated that the Ag nanoparticles were effectively stabilized and reduced by the alcohol-modified ionic surfactants. Thin films of Ag nanoparticles solution in water were drop cast onto a 300 mesh carbon supported film copper grid.

Bright field images and normal incidence selected area electron diffraction (SAED) patterns were obtained using the Phillips CM-120 high resolution transmission electron microscope (HRTEM) at 200keV beam energy to determine the average particle size and distribution and collected in Figure 2. UV-visible absorption spectra were recorded with an SCINCO S3100 UV-visible spectrophotometer with a 1-cm quartz cell.

#### 3.1.4. MIC measurements

As test strains seven microorganisms were chosen: *Bacillus subtilis* KCTC1914, *Staphylococcus aureus* 209 KCTC1916, and antibiotic resistant S*taphylococcus aureus* R209 KCTC1928 among gram positive bacteria, *Escherichia coli* KCTC1924, *Salmonella.thypimurium* KCTC1940, among gram negative bacteria, and *Candida albicans* KCTC1940 as a representative fungus.

The MIC determination tests were carried out by the automatic serial dilution method in LB broth (microtitre). Bacterial and fungal inocula were prepared by dilution of an overnight broth culture to give the equivalent of approximately 10^6^ cell/mL [26]. The efficiency of these Ag nanoparticles were evaluated against bacterial and fungal strains through measurements of minimal inhibitory concentrations (MIC) expressed in μg/mL, values after one day of exposure are shown in Table 1.

## Figures and Tables

**Table 1.** The MIC of silver nanoparticles solution stabilized by hydroxyl functionalized cationic surfactants.

Compound number	Substituents			Anions	MIC (μg/mL)					
	R_1_	R_2_	R_3_		Tested organisms (bacteria and fungi)					
					ES	ST	SA	SAR	BS	CA
**1a**	C_14_H_29_	H	HEt	Br	<4	<4	<4	<4	<4	<4
**2a**	C_14_H_29_	H	diHPr	Cl	39	39	19	39	19	19
**3a**	C_12_H_25_	Me	HEt	Cl	39	156	156	78	78	78
**3b**	C_14_H_29_	Me	HEt	Cl	78	156	78	39	19	9
**3c**	C_16_H_33_	Me	HEt	Cl	10	10	19	19	10	10
**BAC**							8	8	8	
**CPC**							8	8	8	
**Gentamycin**					1	0.5	0.25	0.25	1	
**Kanamycin**					16	1	2	1	2	

BAC: benzalconium chloride;

CPC: cetylpyridinium chloride;

ES: E.coli KCTC1924;

ST: S.typhimurium KCTC1926;

SA:S.aureus 209 KCTC1916;

SAR:S.aureus R209 KCTC1928;

BS:B.subtilis KCTC1914;

CA:C.albicans KCTC1940

GM; Gentamycin;

KM:Kanamycin.Me:CH_3_;

HEt:Hydroxyethyl=HOCH_2_CH_2_;

diHPr:dihydroxypropyl=CH_2_CH_2_(OH)CH_2_(OH).

**Table 2.** ^1^H-NMR chemical shift data for 1-tetradecyl-3-hydroxyethylimidazolium bromide.

No	Im:H^2^	Im:H^5^	Im:H^4^	CH_2_O	NCH_2_	NCH_2_	CH_2_	γ	mc	ω
**1a**	9.03, s	7.64, d	7.58, d	4.27–4.38, m	3.94–3.95, m	3.82–3.84, t	3.66–3.68, t	1.89–2.07, m	1.27–1.34, br.s	0.83–0.86, t

**Table 3.** ^1^H-NMR chemical shift data for 1-tetradecyl– 3-(2’,3’-dihydroxy)propylimidazolium chloride.

No	Im:H^2^	Im:H^5^	Im:H^4^	CH_2_O	OCH_2_	OCH	NCH_2_	CH_2_	mc	ω
**2a**	9.02, s	7.66–7.69, d	7.03–7.26, d	4.31–4.48, d	4.04–4.06, d	3.92–3.94, m	3.62–3.75, m	1.79–1.92, m	1.31, br. s	0.89-0.93, t

**Table 4.** ^1^H-NMR chemical shift data for long chain quaternary 1-alkyl-3-hydroxyethyl-2-methylimidazolium chlorides.

No	Im:H^5^	Im:H^4^	CH_2_O	OH	NCH_2_	αNCH_2_	C^2^CH_3_	βCH_2_	γCH_2_	mc	ω
**3a**	7.42	7.19	4.28	4.16	3.92	3.79	2.78	2.53	1.32	1.26	0.85
**3b**	7.45	7.21	4.31	4.19	3.93	3.68	2.81	2.56	1.35	1.28	0.87

**Table 5.** FAB-MS data.

**Comp No**	**1a**	**2a**	**3a**	**3b**
**m/z**	309.04	339.04	323.2	351.2
